# Overexpression of MdZAT5, an C2H2-Type Zinc Finger Protein, Regulates Anthocyanin Accumulation and Salt Stress Response in Apple Calli and *Arabidopsis*

**DOI:** 10.3390/ijms23031897

**Published:** 2022-02-08

**Authors:** Da-Ru Wang, Kuo Yang, Xun Wang, Xiao-Lu Lin, Lin Rui, Hao-Feng Liu, Dan-Dan Liu, Chun-Xiang You

**Affiliations:** 1 National Key Laboratory of Crop Biology, National Research Center for Apple Engineering and Technology, College of Horticulture Science and Engineering, Shandong Agricultural University, Taian 271018, China; 15953327292@163.com (D.-R.W.); kuokuoy@163.com (K.Y.); wx20145015@126.com (X.W.); smileboy2021@163.com (L.R.); haofengliu3@163.com (H.-F.L.); 2 College of Plant Protection, Shandong Agricultural University, Taian 271018, China; 15621322802@163.com; 3 College of Agriculture, Yunnan University, Kunming 650091, China

**Keywords:** apple, MdZAT5, anthocyanin, salt tolerance

## Abstract

Zinc finger proteins are widely involved and play an important role in plant growth and abiotic stress. In this research, *MdZAT5*, a gene encoding C2H2-type zinc finger protein, was cloned and investigated. The *MdZAT5* was highly expressed in flower tissues by qRT-PCR analyses and GUS staining. Promoter analysis showed that *MdZAT5* contained multiple response elements, and the expression levels of *MdZAT5* were induced by various abiotic stress treatments. Overexpression of *MdZAT5* in apple calli positively regulated anthocyanin accumulation by activating the expressions of anthocyanin biosynthesis-related genes. Overexpression of *MdZAT5* in *Arabidopsis* also enhanced the accumulation of anthocyanin. In addition, *MdZAT5* increased the sensitivity to salt stress in apple calli. Ectopic expression of *MdZAT5* in *Arabidopsis* reduced the expression of salt-stress-related genes (*AtNHX1* and *AtABI1*) and improved the sensitivity to salt stress. In conclusion, these results suggest that *MdZAT5* plays a positive regulatory role in anthocyanin accumulation and negatively regulates salt resistance.

## 1. Introduction

Among the numerous external natural environmental factors, light (one of the most important) affects the entire plant life cycle [1,2,3]. Light is an essential factor in anthocyanin synthesis [4]. Previous studies have shown that the longer the light exposure and the greater the light intensity, the more conducive to anthocyanin formation [5,6]. Additionally, salt is one of the most important environmental stresses, which limits the growth and development of plants and poses a serious threat to global agriculture [7,8]. Salt stress significantly inhibits the growth of apple plants, mainly manifesting in slow growth, leaf wilting, and other symptoms, which even leads to plant death in serious cases [9]. Salt tolerance varies amongst different apple varieties. For example, Red fruit Begonia is affected by salt stress at 0.5 mg/g NaCl, and *Mulus zhumei* and *Midget Crabapple* appear to be affected by salt stress at 5.5 mg/g NaCl [10]. In order to adapt to complex and changing environmental factors, complex regulatory mechanisms have gradually evolved in plants [11,12,13]. At present, many transcription factors have been found to regulate anthocyanin accumulation and respond to salt stress, such as MYB, bHLH, WRKY, ZFP, DREB/CBF, NAC, and AP2/ERF [14,15,16,17,18,19,20,21]. 

Zinc finger proteins are among the most widely distributed proteins in eukaryotes [22,23]. The Cys2/His2 (C2H2)-type zinc finger proteins, also known as TFIIIA zinc finger protein, are the most common protein in the zinc finger gene family [23]. TFIIIA transcription factor was first found in *Xenopus laevis* [24]. In plants, C2H2-type zinc finger proteins have a highly conserved amino acid sequence (QALGGH) located at the junction of the zinc finger and the DNA α Spiral zone [25]. In addition to the typical zinc finger domain that binds to DNA, most C2H2-type zinc finger proteins also contain three characteristic motifs [17,26]. One is the ERF-associated amphiphilic repression (EAR) motif (also known as DLN-box)—a short hydrophobic transcriptional repressor domain near the C-terminal. The second is a nuclear localization signal (NLS)—mainly related to subcellular localization. The third is an L-box—possibly related to protein interactions.

The C2H2-type zinc finger proteins play an important role in the growth and development of plants [27,28,29]. In *Arabidopsis*, JAGGED (*JAG*) can regulate cell differentiation and flower morphological development [30]. ZINC FINGER of *ARABIDOPSIS THALIANA* 6 (*ZAT6*) plays an important role in the regulation of anthocyanin under hydrogen peroxide treatment [18]. ZINC FINGER PROTEIN 5 (*ZFP5*) can affect root hair development by directly regulating root hair development-related genes [31]. In addition, it also plays an important role in abiotic stress [32,33]. *AtZAT18* positively regulates plant drought tolerance, while its mutant decreased the tolerance to drought stress in *Arabidopsis* [34]. *Triticum aestivum* predicted that Dof zinc finger protein (*TaZNF*) regulates salt tolerance [35]. Ectopic expression of *GmZFP3* in *Arabidopsis* plays a negative regulatory role in drought response [36]. *Oryza sativa* drought-responsive zinc finger protein 1 (*OsDRZ1*) overexpression in rice can improve plant drought tolerance by accumulating more proline and scavenging ROS [37]. A multi-stress-responsive gene (*OsMSR15)* in *Oryza sativa* L. shows positive regulation in cold, drought, and heat stress conditions at various developmental stages of rice [38]. MdZAT10 positively regulates JA-induced leaf senescence by interacting with MdBT2 (BTB-TAZ 2) and negatively regulates plant drought tolerance in apple [39,40].

So far, C2H2-type zinc finger proteins have been widely cloned and identified in *Arabidopsis*. However, *ZAT5* has been rarely reported in other species, and its function in apple is poorly understood. In this assay, we identified a C2H2-type zinc finger protein transcription factor MdZAT5 in apple and characterized its roles in regulating apple calli and transgenic *Arabidopsis* anthocyanin accumulation and salt stress.

## 2. Results

### 2.1. Identification and Bioinformatics Analysis of the MdZAT5 Gene in Apple

**Figure 1:**
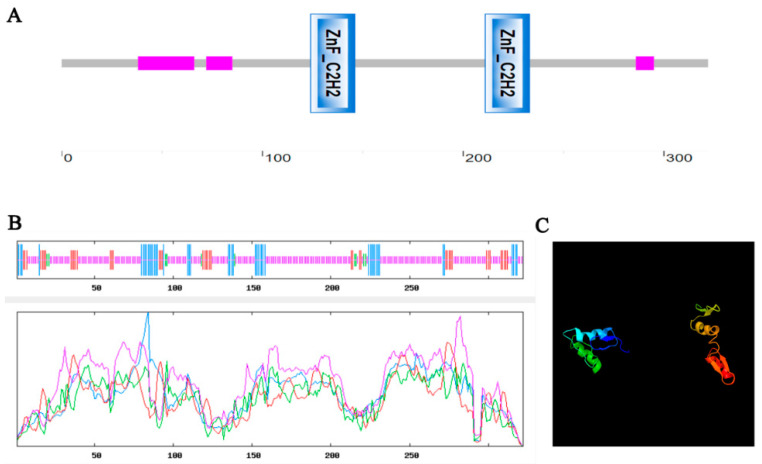
Basic information about the *MdZAT5* sequence. (**A**) Conserved sequence of MdZAT5 protein. The blue rectangle indicates the zinc finger domain. The numbers represent the length of amino acids. (**B**,**C**) predicted the secondary and tertiary protein structures of MdZAT5, respectively. The numbers denote the length of amino acids.

*MdZAT5* (MD03G1128800) was identified as the closest apple homolog of the C2H2-type zinc finger transcription factor *AtZAT5* (At2G28200) on the NCBI database. Its full-length cDNA was 969 bp long and encoded 322 aa. As shown in Figure 1A, it contained two conserved zinc finger domains. We predicted the secondary structure of MdZAT5 protein and found that it was mainly random coils (69.88%), followed by alpha-helices (14.29%), extended-strands (12.73%), and beta-turns (3.11%) (Figure 1B). Based on the prediction results of the secondary structure, the tertiary structure was predicted (Figure 1C).

### 2.2. Phylogenetic and Conserved Motif Analysis of ZAT5 Proteins from Different Plants

**Figure 2:**
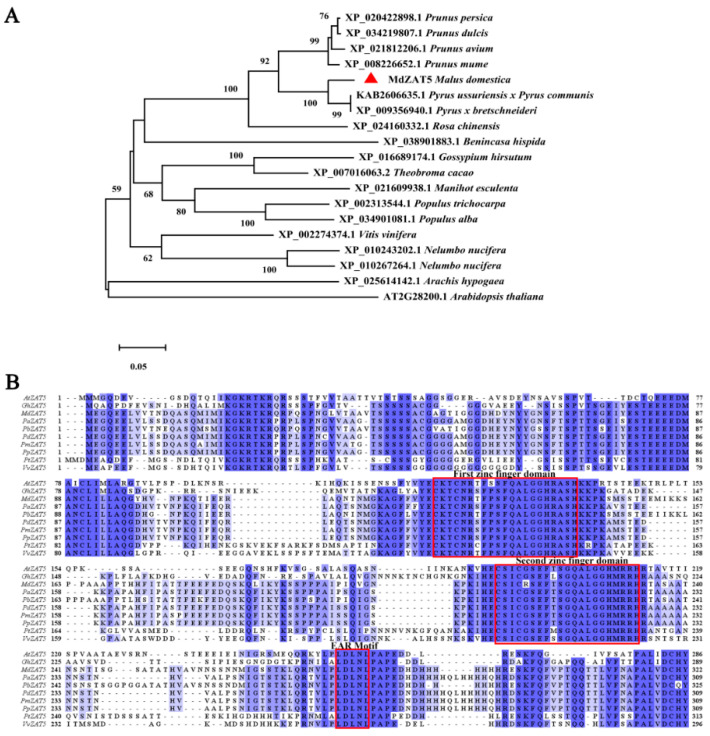
Phylogenetic tree, amino acid sequence alignment, and conserved motifs analysis. (**A**) The phylogenetic tree of ZAT5 proteins from 18 different plants. (**B**) Comparison of amino acid sequences of ZAT5 proteins from 10 different plants. The red triangle represents MdZAT5. The red box represents a conserved domain. They all have two conserved zinc finger domains and an EAR motif.

In order to analyze the phylogenetic relationship between MdZAT5 protein and ZAT5 proteins of other species, an evolutionary tree was constructed using MEGA_X software. It was found that the ZAT5 protein of apple (*Malus domestica*) showed the closest evolutionary relationship with pear (*Pyrus* × *bretschneideri* and *Pyrus ussuriensis* × *Pyrus communis*) (Figure 2A). In addition, we compared the ZAT5 protein sequences of apple and the other nine species and found that they all contained two conserved zinc finger domains and an EAR motif (Figure 2B).

### 2.3. Tissue Expression Pattern of MdZAT5

**Figure 3:**
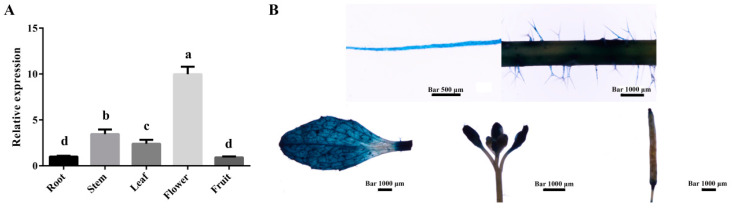
Tissue expression analysis of *MdZAT5*. (**A**) The relative expression level of *MdZAT5* in different tissues (roots, stems, leaves, flowers, and fruits) by qRT-PCR. (**B**) Tissue expression analysis of *MdZAT5* via GUS staining in transgenic *Arabidopsis*. Different lowercase letters represent a significant difference (*p* < 0.05). Data are the mean ± SD of three independent replicates.

To further explore the potential biological function of *MdZAT5* in apple, we detected its expression in five different organ tissues of apple (roots, stems, leaves, flowers, and fruits) by qRT-PCR. *MdZAT5* was expressed in organs and tissues, among which the expression was the highest in flowers, followed by stems and leaves, and lower in roots and fruits (Figure 3A). The constructed *ProMdZAT5::GUS* vector was genetically transformed into *Arabidopsis* to obtain *ProMdZAT5::GUS* transgenic *Arabidopsis*. GUS staining showed that *MdZAT5* was expressed in roots, stems, leaves, flowers, and fruits (Figure 3B), and the results were consistent with Figure 3A.

### 2.4. Cis-Elements Analysis of MdZAT5 Promoter Sequence and Expression Patterns of MdZAT5

We analyzed *MdZAT5 cis*-elements in promoter 2000 bp upstream using the PlantCARE online software. There are various response elements in the *MdZAT5* promoter (Table 1), such as *cis*-elements in response to plant hormones: abscisic responsive element (ABRE) and MeJA responsive element (CGTCA-motif). A large number of light response elements is known in the *MdZAT5* promoter, such as Box 4, G-Box, GATA-motif, GT1-motif, MRE, and TCCC-motif. In addition, there is an ARE element that responds to hypoxia in the *MdZAT5* promoter.

**Figure 4:**
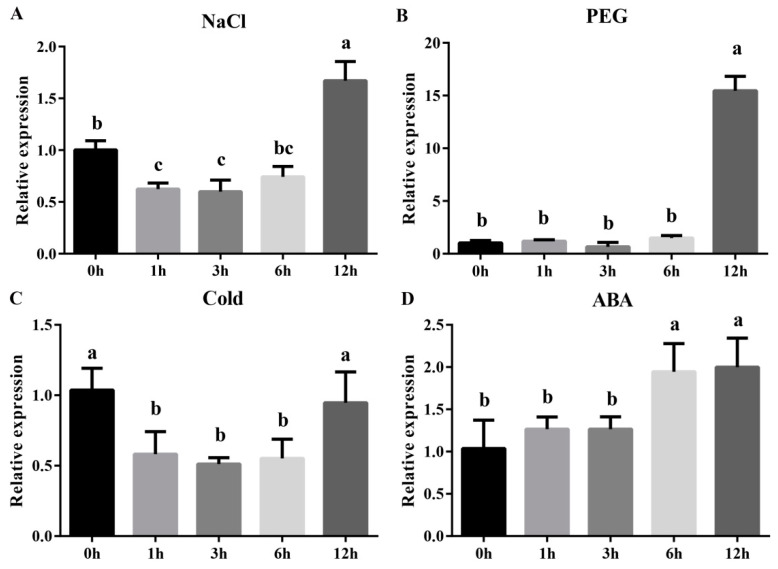
The expression pattern of *MdZAT5* under different treatment conditions. The relative expression of *MdZAT5* in 150 mM NaCl (**A**), 10% PEG6000 (**B**), 4 °C (**C**), and 100 μM ABA (**D**), respectively. Different lowercase letters represent a significant difference (*p* < 0.05). Data are the mean ± SD of three independent replicates.

To gain further insight into the expression patterns of *MdZAT5* with multiple abiotic stressors, we measured the expression of the *MdZAT5* gene under NaCl (150 mM), PEG6000 (10%), temperature (4 °C), and ABA (100 μM) treatments. Under treatment with 150 mM NaCl, the expression of *MdZAT5* reached its highest at 12 h, and the overall trend was first decreasing and then increasing (Figure 4A). In response to 10% PEG 6000, the expression of *MdZAT5* reached its highest at 12 h (Figure 4B). Under low temperature conditions (4 °C), *MdZAT5* showed a downward trend first and then an upward trend, reaching its maximum at 12 h (Figure 4C). The expression of *MdZAT5* showed an upward trend with the treatment of 100 μM ABA over time (Figure 4D). These results showed that *MdZAT5* expression was caused by different stressors, which meant that *MdZAT5* played an important regulatory role in the process of stress response.

### 2.5. The Abiotic Stress Response of MdZAT5

**Figure 5:**
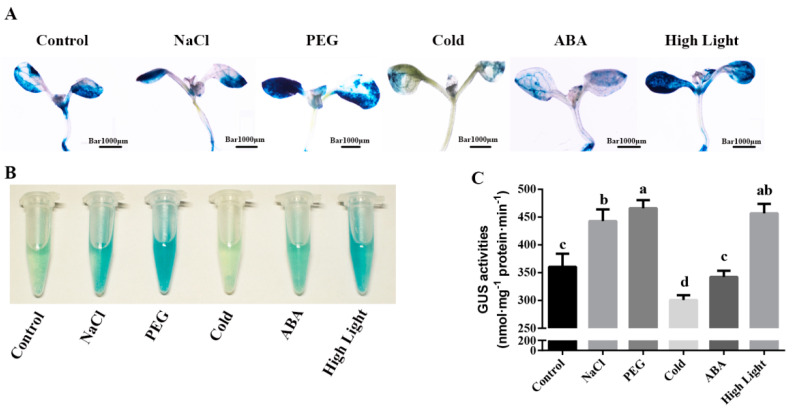
GUS staining in *ProMdZAT5::GUS* transgenic calli and *Arabidopsis*. The *ProMdZAT5::GUS* transgenic *Arabidopsis* (**A**) and calli (**B**), treated with 24 °C, 150 mM NaCl, 6% PEG, 4 °C, 100 μM ABA, and high light. (**C**) The GUS activity of *MdZAT5* of (**B**). Different lowercase letters represent a significant difference (*p* < 0.05). Data are the mean ± SD of three independent replicates.

Based on the analysis of *cis*-acting elements and expression patterns, we treated *ProMdZAT5::GUS* transgenic *Arabidopsis* with different treatment conditions. Compared with the control, 150 mM NaCl, 6% PEG6000, and high light could significantly promote GUS activity, but at 4 °C, it was lightly stained. Moreover, we found that 100 μM ABA caused no significant changes in leaf color relative to the control (Figure 5A). In addition, we also observed that the staining was deeper in older leaves and less intense in younger leaves, indicating that *MdZAT5* was expressed higher with leaf age. We obtained *ProMdZAT5::GUS* transgenic calli, and their staining results were consistent with that of *ProMdZAT5::GUS* transgenic *Arabidopsis* (Figure 5A–C).

### 2.6. Overexpression of MdZAT5 in Apple Calli and Arabidopsis Promoted the Accumulation of Anthocyanin

**Figure 6:**
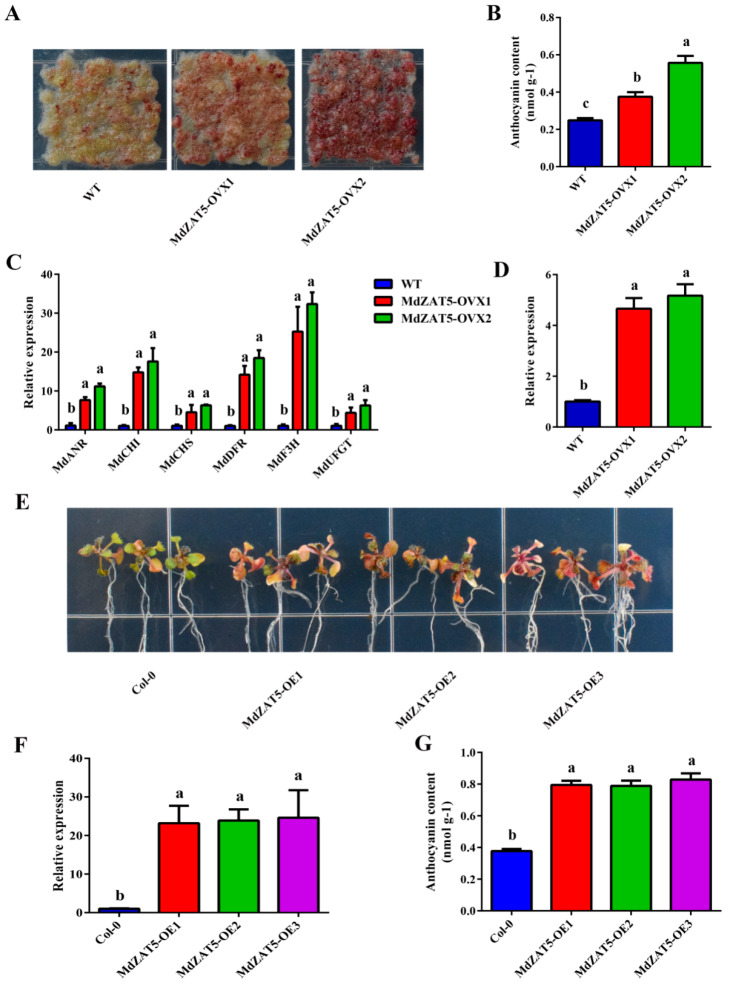
Overexpression of *MdZAT5* in apple calli and *Arabidopsis* promoted anthocyanin accumulation. The phenotypes (**A**) and anthocyanin content (**B**) of WT and *MdZAT5-OVX*. Expression analysis of *MdZAT5* (**D**) and genes involved in anthocyanin biosynthesis-related genes (*MdANR*, *MdCHI*, *MdCHS*, *MdDFR*, *MdF3H,* and *MdUFGT*) (**C**) in WT and *MdZAT5-OVX*. The phenotypes (**E**) and anthocyanin content (**G**) of Col-0 and *MdZAT5-OE*. (**F**) Expression analysis of *MdZAT5* in Col-0 and *MdZAT5-OE*. Different lowercase letters represent a significant difference (*p* < 0.05). Data are the mean ± SD of three independent replicates.

To study the function of *MdZAT5*, we constructed the *MdZAT5* overexpression vector and transferred it into apple calli. Wild-type calli (WT) and transgenic apple calli (*MdZAT5-OVX*) were cultured under high light for 18 days. As shown in Figure 6A, *MdZAT5-OVX* accumulated more anthocyanins, while WT accumulated less anthocyanins. At the same time, we used a spectrophotometer to quantitatively determine the content of anthocyanin. The results also showed that the anthocyanin content of *MdZAT5-OVX* was higher than that of WT (Figure 6B). In addition, the expression levels of flavonoid structural genes in WT and *MdZAT5-OVX* were analyzed by qRT-PCR. The results showed that compared with WT, the expression levels of anthocyanin biosynthesis-related genes (*MdANR*, *MdCHI*, *MdCHS*, *MdDFR*, *MdF3H,* and *MdUFGT*) increased by different degrees (Figure 6C).

In addition, we genetically transformed the constructed *MdZAT5* overexpression vector into *Arabidopsis* (Columbia ecotype) and obtained three overexpression lines (OE1, OE2, and OE3) (Figure 6F). Comparing the anthocyanin content of Col-0 and *MdZAT5-OE*, it was found that under high light, the anthocyanin content accumulated by the three overexpression lines was significantly higher than that of Col-0 (Figure 6G).

### 2.7. MdZAT5 Increased Sensitivity to Salt Stress in Transgenic Apple Calli and Arabidopsis

**Figure 7:**
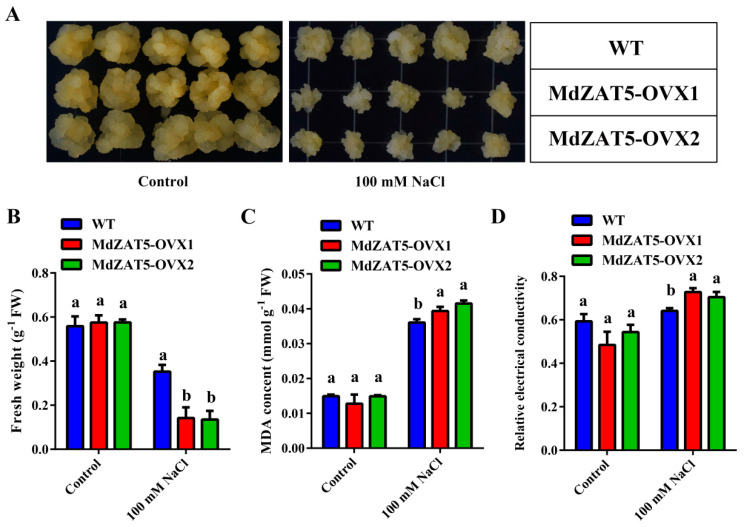
*MdZAT5* enhanced the sensitivity to salt in apple calli. (**A**) The phenotypes of WT and *MdZAT5-OVX* with 100 mM NaCl. Fresh weight (**B**), MDA content (**C**), relative electronic conductivity (**D**) of WT, and *MdZAT5-OVX*. Different lowercase letters represent a significant difference (*p* < 0.05). Data are the mean ± SD of three independent replicates.

To further explore the function of *MdZAT5* under abiotic stress, the 16-day-old WT and *MdZAT5-OVX* were transferred to 100 mM NaCl MS medium. As shown in Figure 7A, the growth rate of *MdZAT5-OVX* was much lower than that of WT in 100 mM NaCl. Meanwhile, the fresh weight of *MdZAT5-OVX* was significantly lower than that of WT, and its MDA content and relative electronic conductivity were significantly higher than that of WT (Figure 7B–D). Therefore, the overexpression of *MdZAT5* negatively regulates the salt resistance of apple calli.

**Figure 8:**
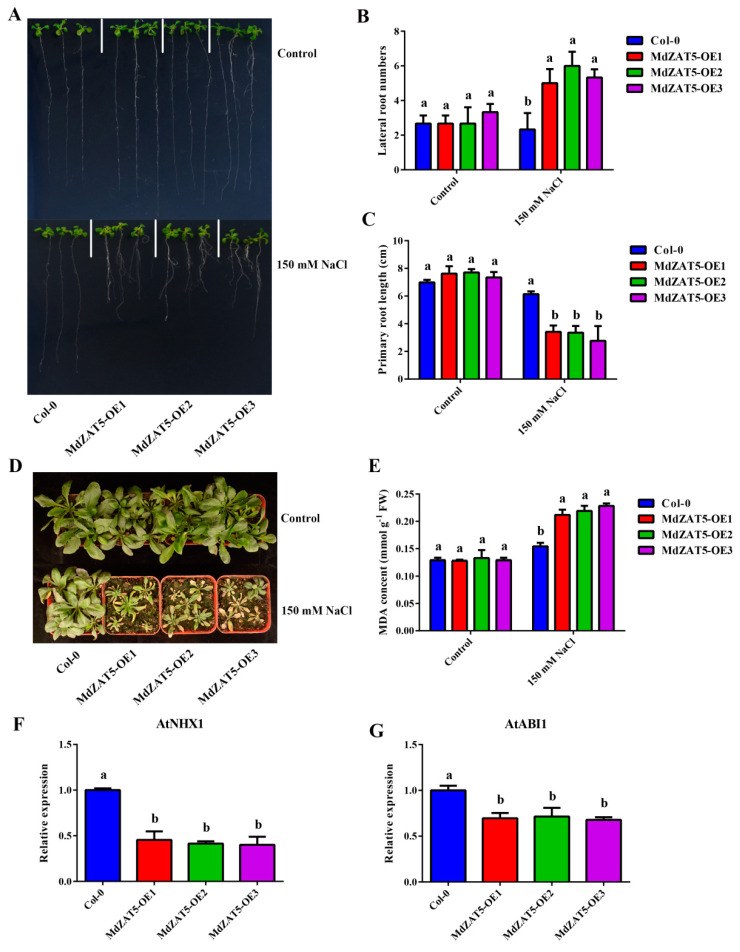
*MdZAT5* enhanced the sensitivity to salt in transgenic *Arabidopsis*. (**A**) The phenotypes of *Arabidopsis* seedlings treated with MS medium, 150 mM NaCl treatment. Lateral root numbers (**B**) and primary root length (**C**) in Col-0 and *MdZAT5-OE*. (**D**) Phenotypes of *Arabidopsis* treated with 150 mM NaCl after 14 days and MDA content (**E**). The expression level of *AtNHX1* (**F**) and *AtABI1* (**G**) in Col-0 and *MdZAT5-OE*. Different lowercase letters represent a significant difference (*p* < 0.05). Data are the mean ± SD of three independent replicates.

Furthermore, *Arabidopsis* seedlings (Col-0 and *MdZAT5-OE*) were grown on MS medium for 3 days and then transferred to 150 mM NaCl MS medium for 14 days. In the control condition, there was no significant difference in the number of lateral roots or primary root lengths. However, the number of lateral roots of *MdZAT5-OE* was greater than that of Col-0, and the length of primary roots was lower than that of Col-0 under the salt treatment (Figure 8B,C). In addition, we conducted salt tolerance tests on Col-0 and transgenic plants for 14 days. As shown in Figure 8D, Col-0 grew normally, while *MdZAT5-OE* had yellow and wilting leaves. Simultaneously, the content of MDA in *MdZAT5-OE* was significantly higher than that in Col-0 plants under salt stress. In order to further study the role of *MdZAT5* in the signal pathway of salt stress, we detected the expression levels of *AtNHX1* and *AtABI1* in *Arabidopsis* by qRT-PCR. The results showed that the expression levels of *AtNHX1* and *AtABI1* in *MdZAT5-OE Arabidopsis* were significantly lower than those in Col-0 (Figure 8F,G).

### 2.8. Ectopic Expression of MdZAT5 Increased ROS Accumulation under Salt Stress

**Figure 9:**
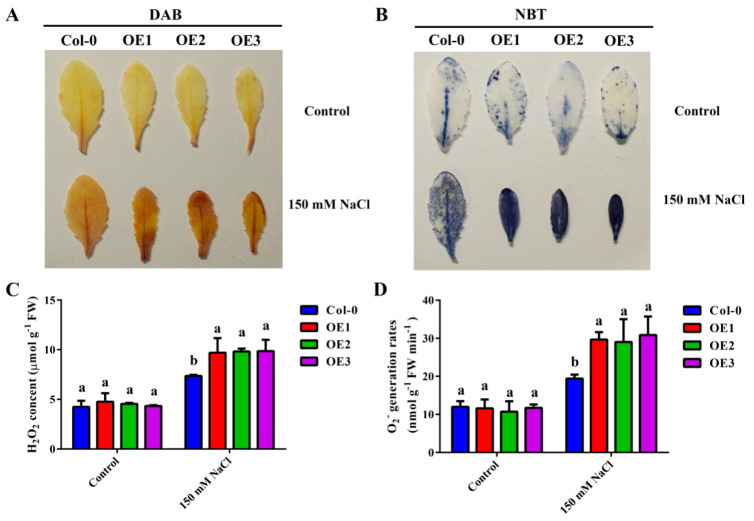
Ectopic expression of *MdZAT5* enhances ROS buildup under salt treatment. DAB staining for H_2_O_2_ (**A**) and NBT staining for O_2_^−^ (**B**) in Col-0 and *MdZAT5-OE Arabidopsis* leaves after 14 days of salt treatment. H_2_O_2_ content (**C**) and O_2_^−^ generation rates (**D**) in Col-0 and *MdZAT5-OE Arabidopsis* after 14 days of salt treatment. Different lowercase letters represent a significant difference (*p* < 0.05). Data are the mean ± SD of three independent replicates.

H_2_O_2_ levels were measured using diaminobenzidine (DAB) staining and O_2_^−^ using nitro blue tetrazolium (NBT). In the control group, no significant difference was observed between WT and *MdZAT5-OE*. Under salt treatment, the staining of *MdZAT5-OE* was deeper, while that of Col-0 was weaker (Figure 9A,B). At the same time, we quantitatively determined H_2_O_2_ and O_2_^−^, and the results were consistent with their staining results. The contents of H_2_O_2_ and O_2_^−^ of *MdZAT5-OE* were significantly higher than those of Col-0 under salt stress, indicating that *MdZAT5* increased sensitivity to salt stress in *Arabidopsis* (Figure 9C,D).

## 3. Discussion

C2H2-type zinc finger proteins played key roles in regulating plant growth, abiotic, and biotic stress responses in plants [37,41,42]. In recent years, many studies have studied the function of C2H2-type zinc finger proteins. Their functions have been widely characterized in *Arabidopsis* and rice. However, little has been reported in apple. Here, we isolated *ZAT5* from apple and found that both *ZAT5* and *AtZAT5* contained two highly conserved zinc finger domains and an EAR motif, indicating that it has the conserved function of this family (Figure 1A and Figure 2B). The EAR motif, a short hydrophobic region, has been shown to function as repressor, e.g., *ZAT6*, *STZ*/*ZAT10*, *ZAT11,* and *ZAT12* in *Arabidopsis* [43,44,45]. Different treatments (salt, drought, cold, ABA, and high light) induced the expression of *MdZAT5*, suggesting that *MdZAT5* may be involved in the tolerance of many abiotic stressors (Figure 4 and Figure 5). Previous studies have shown that *AtSTZ*/*ZAT10* is induced by the same treatments [44,46]. *MdZAT5* transgenic apple calli and *Arabidopsis* were confirmed to perform biological functions. In this study, *MdZAT5* was involved in anthocyanin synthesis and salt stress response in apple.

Anthocyanin, a polyphenol water-soluble plant pigment, exists widely in flowers, fruits, stems, leaves, and seeds of plants [47]. Here, the expression of *MdZAT5* was highest in flowers (Figure 3A). We analyzed promoter sequences of *MdZAT5* and found a large number of light-responsive elements (Table 1). Light is the most important external factor regulating anthocyanin synthesis [4]. *ProMdZAT5::GUS* transgenic *Arabidopsis* was deeply up-regulated under high light treatment, consistent with the result of GUS staining of *ProMdZAT5::GUS* transgenic calli (Figure 5). Under high light, overexpression of *MdZAT5* actively regulated anthocyanin accumulation in apple calli and *Arabidopsis*, further indicating that *MdZAT5* plays an important role in plant response and adaptation to high light. Under high light, *AtZAT12* promotes the increase of anthocyanin and chlorophyll content [48]. In Petunia, *ZPT2-1* participates in anthocyanin synthesis [49]. Few studies have reported the functions of zinc finger proteins in the regulation of anthocyanin synthesis; thus, more work is needed in this area to perfect the content.

Salt stress is one of the most important limiting factors in plant growth, development, and yield [50,51]. Therefore, it is necessary to study salt-stress-related genes and their functions to improve crops. Previous studies have reported that many C2H2-type zinc finger proteins are involved in the regulation of salt stress as transcriptional activators or inhibitors [52]. In *Arabidopsis*, SALT-INDUCED ZINC FINGER PROTEIN1 (*AtSIZ1*) positively regulates salt tolerance by maintaining osmotic balance and ion homeostasis [53]. RING/FYVE/PHD ZFP (*AtRZFP*) enhances salt and osmotic tolerance by scavenging ROS, maintaining Na^(+)^ and K^(+)^ homeostasis [54]. *AtZFP3* can enhance the salt resistance of *Arabidopsis*, and its expression level is inhibited under salt stress [55]. OsZFP213 interacts with OsMAPK3 to improve salt tolerance by scavenging reactive oxygen [56]. In other species, there are also reports of C2H2-type zinc finger proteins involved in salt stress, such as wheat, soybean, tomato, and sweet potato [35,57,58,59]. 

In this study, *ProMdZAT5::GUS* transgenic *Arabidopsis* and calli also further confirmed that *MdZAT5* was related to salt stress (Figure 5). Under salt stress, transgenic *MdZAT5* apple calli and *Arabidopsis* showed weaker growth than wild-type plants, indicating that *MdZAT5* plays a negative regulatory role in plant response and adaptation to salt stress (Figure 7 and Figure 8). We measured the content of MDA in apple calli and *Arabidopsis*, and found that the MDA content of transgenic plants was higher than in those of WT plants (Figure 7C and Figure 8E). The content of MDA can reflect the degree of stress damage to plants [60]. The accumulation of ROS was related to the content of MDA [61]. Deeper levels of DAB/NBT staining, higher H_2_O_2_ content levels, and higher O_2_^−^ generation rates under salt treatment indicate that more ROS accumulation occurred in transgenic plants (Figure 9). In addition, maintaining ion balance is an important method for plants to resist salt stress [62]. *NHX1*, a Na^+^/H^+^ antiporter located on the vacuolar membrane, plays an important role in maintaining ion homeostasis in plant cells [63]. *ABI1* is a salt-stress-related gene [64]. The *AtNHX1* and *AtABI1* expression levels in the overexpressed *MdZAT5 Arabidopsis* were significantly lower than those in Col-0 (Figure 8F,G). The above results show that overexpression of *MdZAT5* enhances the sensitivity of plants to salt stress by reducing the expression level of *NHX1* and *ABI1*.

In this study, we identified a novel *MdZAT5* transcription factor that directly or indirectly activates the expression of anthocyanin synthesis-related genes to increase anthocyanin accumulation or reduce the expression of salt-stress-related genes to improve the sensitivity of salt stress. This study provides new insights for future research on anthocyanin accumulation and resistance to salinization and provides new candidate genes for improving apple quality and abiotic stress. However, the potential mechanism of *MdZAT5* in regulating anthocyanin accumulation and salt stress is not clear. We next intend to further improve its regulatory mechanism by verifying the direct downstream protein or gene targets of *MdZAT5*. 

## 4. Materials and Methods

### 4.1. Plant Materials and Growth Conditions

The apple (*Malus* × *domestica* ‘Royal Gala’) shoot cultures were stored at 25 °C on MS solid medium containing 0.5 mg/L 6-benzylaminopurine (6-BA) and 0.5 mg/L naphthyl acetic acid (NAA) for a 16/8 h light/dark photoperiod and subcultured at 30-day intervals. To obtain self-rooted plantlets, the 3-week-old shoot cultures were transferred to a root-inducing MS solid medium containing 0.2 mg/L indoleacetic acids (IAA). For tissue expression analysis, the roots, stems, leaves, flowers, and fruits were collected 80 days after flowering from 7-year-old ‘Gala’ apple tree (Taian, China). Four-week-old self-rooted apple seedlings were treated with NaCl (150 mM), PEG 6000 (10%), temperature (4 °C), and ABA (100 μM), as described in [45]. 

Apple calli from the ‘Orin’ cultivar were grown on MS solid medium of 1.5 mg/L 2, 4-dichlorophenoxyacetic acid (2, 4-D), and 0.4 mg/L 6-BA for 18 days in the dark at 24 °C. Then, for the stress treatment, calli were cultured in a medium with high light and NaCl (100 mM) for 18 days. The seeds of ecotype Columbia (Col-0) and transgenic *Arabidopsis (MdZAT5-OE1*, *MdZAT5-OE2*, *MdZAT5-OE3*) were sterilized and vernalized for 3 days at 4 °C. Then, *Arabidopsis* seedlings were sown on MS solid medium for 3 days under a photoperiod of 16/8 h light/dark and transferred to solid medium with high light (~300 μmol m^−2^ s^−1^) and NaCl (150 mM) for 7 and 14 days, respectively. Twenty-one-day-old seedlings of *Arabidopsis* were supplied with 150 mM NaCl for 14 days in soil.

### 4.2. Bioinformatics Analysis of the MdZAT5 Gene

The basic information of the MdZAT5 sequence came from the NCBI database (https://www.ncbi.nlm.nih.gov/, accessed on 3 November 2020). The secondary and tertiary structure prediction of MdZAT5 adopted SOPMA (https://npsa-prabi.ibcp.fr/cgi-bin/npsa_automat.pl?page=npsa_sopma.html, accessed on 15 March 2021) and Phyre2 (http://www.sbg.bio.ic.ac.uk/~phyre2/html/page.cgi?id=index, accessed on 15 March 2021), respectively [65].

### 4.3. Phylogenetic Analysis and Multiple Sequence Alignment of ZAT5 Proteins

The adjacency algorithm of the online software MEGA_X was used to construct ZAT5 evolutionary neighbor-joining trees of different plants (the step test was set to 1000 times, substitution method was the Poisson model) [66]. A multiple sequence alignment of ZAT5 proteins from 10 different plants was performed using Clustal Omega. We found several highly conserved domains, which were visualized by the online software Jalview.

### 4.4. Analysis of the MdZAT5 Promoter

The cis-element in the *MdZAT5* promoter (2000 bp upstream of the transcription initiation site) was analyzed with the online software PlantCARE (http://bioinformatics.psb.ugent.be/webtools/plantcare/html/, accessed on 18 March 2021) [67].

### 4.5. RNA Extraction and qRT-PCR Assays

Total RNAs of plant materials, including apple and *Arabidopsis*, were isolated using the RNA Plant Plus Reagent Kit (Tiangen Biotech, Beijing, China). Reverse transcription was conducted for single-stranded DNA synthesis using the PrimScript™ First Strand cDNA Synthesis Kit (TaKaRa, Dalian, China), per the manufacturer’s protocol. qRT-PCR was performed on the extracted RNA using an ABI7500, in which *18S* (apple) and *AtACTIN* (*Arabidopsis*) were used as internal control. Then, relative gene expression analysis was conducted using the cycle threshold (Ct) 2^−ΔΔCT^ method. Quantitative primers are listed in Supplementary Table S1.

### 4.6. Construction of the Expression Vectors and Genetic Transformation

To construct the overexpression vector of *MdZAT5*, we cloned the full-length coding sequence of *MdZAT5* into the plant expression vector pRI101 [40]. The *MdZAT5* (2000 bp promoter fragment from start codon) was cloned into a p1300-GN vector to construct *ProMdZAT5::GUS*, and the vector drives Gus (β-glucuronidase) reporter gene [64]. The *MdZAT5* overexpression vector and *ProMdZAT5::GUS* constructs were transformed into *Arabidopsis* by the flower dipping method to obtain transgenic plants [68]. The calli of transgenic apple were obtained by the Agrobacterium-mediated method [69]. Transgenic *Arabidopsis* and calli were identified with kanamycin.

### 4.7. GUS Histochemical Staining

The 18-day-old transgenic apple calli and 7-day-old transgenic *Arabidopsis* seedlings of *ProMdZAT5::GUS* were cultured on solid medium with NaCl (150 mM), PEG 6000 (10%), temperature (4 °C), ABA (100 μM), and high light (~300 μmol m^−2^ s^−1^) for 24 h. Apple calli or *Arabidopsis* seedlings were subjected to staining using GUS staining buffer (containing 0.5 mM ferrocyanide, 0.1% Triton X-100, 0.1 mM EDTA, 0.5 mM ferricyanide, and 1 mM X-Gluc) and then decolorized with absolute ethanol for 12 h [70].

### 4.8. Measurements of Anthocyanin, Relative Electronic Conductivity, and MDA

The anthocyanin of plants was extracted by the methanol hydrochloric acid method [71]. The plant materials were placed in anthocyanin extract (95% ethanol: 1.5 M HCl = 85:15, v/v) in the dark at room temperature for 24 °C. The absorbance value of the extracted samples was measured at 530, 620, and 650 nm with a UV–Vis spectrophotometer. The anthocyanin content was calculated according to the previous method [72].

The relative electronic conductivity content of apple calli was measured by this method and a DDS-12 conductometer (Hangzhou Wanda Instrument Factory, Hangzhou, China) [73]. The content of MDA in apple calli and *Arabidopsis* was determined by the thiobarbituric acid (TBA)-based method [74].

### 4.9. Measurement of ROS

The H_2_O_2_ content and O_2_^−^ production rate were determined using a kit (Keming, Suzhou, China). H_2_O_2_ level was detected by diaminobenzidine (DAB) histochemical staining, and O_2_^−^ level was detected by nitro blue tetrazolium (NBT) staining in accordance with the methods described in [75].

### 4.10. Statistical Analysis

Each experiment was repeated at least three times (biological repetitions). Each biological repetition was performed at least three times (technical repetitions). All Data processing system (DPS) was used to analyze the significance of the data [76].

## 5. Conclusions

In short, overexpression of *MdZAT5* promotes the expressions of anthocyanin biosynthesis-related genes to actively regulate anthocyanin synthesis and increase sensitivity to salt stress by regulating the expression of *NHX1* and *ABI1*. Our study provides new insight into *MdZAT5*-mediated anthocyanin synthesis and salt resistance and is helpful to further clarify the mechanism of anthocyanin synthesis and salt stress.

## Figures and Tables

**Table 1:** *Cis*-elements analysis of *MdZAT5* promoter regions.

Cis-Element Name	Cis-Element Sequence (5′-3′)	Function	Location
ABRE	ACGTG	*cis*-acting element involved in the abscisic acid responsiveness	+550
CGTCA-motif	CGTCA	*cis*-acting regulatory element involved in the MeJA-responsiveness	−1649
ARE	AAACCA	*cis*-acting regulatory element essential for the anaerobic induction	+1006
Box 4	ATTAAT	part of a conserved DNA module involved in light responsiveness	+245
G-Box	CACGTG	*cis*-acting regulatory element involved in light responsiveness	+550
GATA-motif	AAGGATAAGG	part of a light-responsive element	−1583
GT1-motif	GGTTAA	light-responsive element	+20
MRE	AACCTAA	MYB binding site involved in light responsiveness	−17

## Data Availability

Not applicable.

## Supplementary Materials

The following are available online at https://www.mdpi.com/article/10.3390/ijms23031897/s1.
