# Supplementary material for: Overexpression of MdZAT5, an C2H2-Type Zinc Finger Protein, Regulates Anthocyanin Accumulation and Salt Stress Response in Apple Calli and Arabidopsis
Source: Int J Mol Sci. 2022 Feb 8;23(3):1897. doi: 10.3390/ijms23031897 (PMC8836528; doi:10.3390/ijms23031897)
Supplement: Supplementary file 1 [file ijms-23-01897-s001.zip › ijms-1562940-supplementary.pdf]

Supplementary Table S1: Primers for quantitative real-time PCR.

**Primer name**	**Sequences (5' to 3')**
MdZAT5(PRI)	F:GTCGACATGGAAGGCCAAGAAGAACTA
MdZAT5(PRI)	R:GGATCCATAATGACAATCCACCAAAGCAGG
MdZAT5(qRT)	F:TCATGATCAAGGGCAAGCGT
MdZAT5(qRT)	R:CCAATAGTACCAGCGCCACA
MdZAT5(p1300-GN)	GTCGACGCCTACTTGATGTGGGCACTTA
MdZAT5(p1300-GN)	GGATCCGCTCACAAACAAAAACCCAGAATTTG
MdANR(qRT)	F:TCAACAAAAGATACCCCCAG
MdANR(qRT)	R:GATAGCTAGCTCGATACATGC
MdDFR(qRT)	F:GTTGAGGGAGATAGGGTTTGAG
MdDFR(qRT)	R:GGTAAATGTAAAACAATAGAGAGG
MdUFGT(qRT)	F:GGAAGTGGTTTTGTCGCCTG
MdUFGT(qRT)	R:CATTATTATTGAGCAACGAACAGC
MdF3H(qRT)	F:GCCGATCACCTACACCGAG
MdF3H(qRT)	R:GTACAAGAAGTGGGAAGGC
MdCHI(qRT)	F:GCTACAAATGCGGTGATAG
MdCHI(qRT)	R:CGCCTCCACTACAACCTCC
MdCHS(qRT)	F:GGCAAGTGCTGTCGGATT
MdCHS(qRT)	R:CCCAAAGAAATAACCACAAG
AtNHX1(qRT)	F:TCTTGCTATTGGTGCCATAT
AtNHX1(qRT)	R:AGGTGTCTCGTCTTGATTC
AtABI1(qRT)	F:CCGTCTCACATCTTCGTCGCTAAC
AtABI1(qRT)	R:CTTTCCCTCCTGCGGCTTCAATC
18S	F: ACACGGGGAGGTAGTGACAA
18S	R: CCTCCAATGGATCCTCGTTA
AtACTIN	F: TTTGGAGCCTGGGACTATGGAT
AtACTIN	R: ACGGGGGAATGGGATGAGAT
